# Identification of Positive Chemotaxis in the Protozoan Pathogen Trypanosoma brucei

**DOI:** 10.1128/mSphere.00685-20

**Published:** 2020-08-12

**Authors:** Stephanie F. DeMarco, Edwin A. Saada, Miguel A. Lopez, Kent L. Hill

**Affiliations:** a Molecular Biology Institute, University of California, Los Angeles, California, USA; b Department of Microbiology, Immunology, and Molecular Genetics, University of California, Los Angeles, California, USA; c California NanoSystems Institute, University of California, Los Angeles, California, USA; University at Buffalo

**Keywords:** *Trypanosoma*, cell-cell interaction, chemoattractants, chemotaxis, parasitology

## Abstract

To complete its infectious cycle, the protozoan parasite Trypanosoma brucei must navigate through diverse tissue environments in both its tsetse fly and mammalian hosts. This is hypothesized to be driven by yet unidentified chemotactic cues. Prior work has shown that parasites engaging in social motility *in vitro* alter their trajectory to avoid other groups of parasites, an example of negative chemotaxis. However, movement of T. brucei toward a stimulus, positive chemotaxis, has so far not been reported. Here, we show that upon encountering Escherichia coli, socially behaving T. brucei parasites exhibit positive chemotaxis, redirecting group movement toward the neighboring bacterial colony. This response occurs at a distance from the bacteria and involves active changes in parasite motility. By developing a quantitative chemotaxis assay, we show that the attractant is a soluble, diffusible signal dependent on actively growing E. coli. Time-lapse and live video microscopy revealed that T. brucei chemotaxis involves changes in both group and single cell motility. Groups of parasites change direction of group movement and accelerate as they approach the source of attractant, and this correlates with increasingly constrained movement of individual cells within the group. Identification of positive chemotaxis in T. brucei opens new opportunities to study mechanisms of chemotaxis in these medically and economically important pathogens. This will lead to deeper insights into how these parasites interact with and navigate through their host environments.

**IMPORTANCE** Almost all living things need to be able to move, whether it is toward desirable environments or away from danger. For vector-borne parasites, successful transmission and infection require that these organisms be able to sense where they are and use signals from their environment to direct where they go next, a process known as chemotaxis. Here, we show that Trypanosoma brucei, the deadly protozoan parasite that causes African sleeping sickness, can sense and move toward an attractive cue. To our knowledge, this is the first report of positive chemotaxis in these organisms. In addition to describing a new behavior in T. brucei, our findings enable future studies of how chemotaxis works in these pathogens, which will lead to deeper understanding of how they move through their hosts and may lead to new therapeutic or transmission-blocking strategies.

## Abstract (precis)

Almost all living things need to be able to move, whether it is toward desirable environments or away from danger. For vector-borne parasites, successful transmission and infection require that these organisms be able to sense where they are and use signals from their environment to direct where they go next, a process known as chemotaxis. Here, we show that Trypanosoma brucei, the deadly protozoan parasite that causes African sleeping sickness, can sense and move toward an attractive cue. To our knowledge, this is the first report of positive chemotaxis in these organisms. In addition to describing a new behavior in T. brucei, our findings enable future studies of how chemotaxis works in these pathogens, which will lead to deeper understanding of how they move through their hosts and may lead to new therapeutic or transmission-blocking strategies.

## INTRODUCTION

A fundamental aspect of virtually all motile organisms is the ability to move in response to a change in the environment. One strategy to do this is through chemotaxis, the movement of an organism toward or away from a chemical cue. In microbial systems, chemotaxis has been best characterized in bacteria and social amoebae, which both employ chemotaxis to locate nutrients and avoid unfavorable environments (1–3). Many bacterial pathogens, in particular, rely on chemotaxis to move toward their desired site of infection (4–7). For protozoan pathogens, which typically must navigate through multiple hosts and a variety of different tissues in each host, chemotaxis has also been hypothesized to be necessary for pathogenesis and transmission (8–13).

Trypanosoma brucei is a protozoan pathogen that causes African sleeping sickness in humans and nagana in cattle. T. brucei is transmitted to a mammalian host through the bite of an infected tsetse fly. In the mammalian host, the parasite first mounts a bloodstream infection before penetrating the blood vessel endothelium to enter the central nervous system, resulting in lethality if not treated (14). T. brucei also infiltrates adipose and dermal tissue, and these extravascular sites represent biologically significant parasite reservoirs that may influence pathogenesis and transmission (15–17). Within the tsetse fly vector, T. brucei must complete an ordered series of directional migrations through specific host tissues in order to be transmitted to a new mammalian host (18). Mechanisms underlying tissue tropisms observed in the mammalian host and insect vector are unknown.

Evidence demonstrating that T. brucei can adjust its motility in response to external cues comes from *in vitro* studies of social motility (SoMo), which occurs in procyclic-form T. brucei (tsetse fly midgut stage) when cultivated on semisolid agarose (19). During SoMo, T. brucei cells assemble into groups that engage in collective motility, moving outward from the point of inoculation to form radial projections. Movement outward is cell density dependent, suggesting a quorum sensing component to the control of motility (20). Furthermore, when parasites in projections sense other T. brucei cells, they actively avoid one another, by either stopping their forward movement or changing their direction of movement, thus exhibiting capacity for negative chemotaxis (19, 21). Additional work revealed that SoMo depends on cAMP signaling in the flagellum (22–24), and recent *in vivo* work has demonstrated that flagellar cAMP signaling is required for T. brucei progression through fly tissues (8). Thus, simply being able to move is not sufficient to complete the transmission cycle, and the combined findings support the idea that T. brucei depends on chemotaxis in response to extracellular signals to direct movement through host tissues. To our knowledge however, positive chemotaxis has not been reported for T. brucei.

Here, we report that T. brucei engaging in SoMo exhibits positive chemotaxis toward Escherichia coli, a behavior we term “BacSoMo.” While T. brucei does not typically interact with E. coli in its natural hosts, it does encounter other bacteria, and E. coli serves as an easy-to-control bacterial sample for use in dissecting chemotaxis *in vitro*. We found that the response is mediated by an active change in parasite motility that occurs at a large distance from the bacteria, indicating response to a chemical cue. Supporting this idea, we show that attraction is mediated by a signal that diffuses through the culture medium and requires actively growing E. coli. Our findings allowed us to begin dissecting cellular behavior that underlies chemotaxis in T. brucei, revealing changes in motility at both the group and individual cell levels. We expect these studies to lead to a deeper understanding of how trypanosomes navigate through the diverse environments encountered during their transmission and infection cycle.

(This research was conducted by Stephanie DeMarco in partial fulfillment of the requirements for a doctor of philosophy degree from the University of California, Los Angeles [25].)

## RESULTS

### Socially behaving T. brucei exhibit chemotaxis toward E. coli.

**FIG 1:**
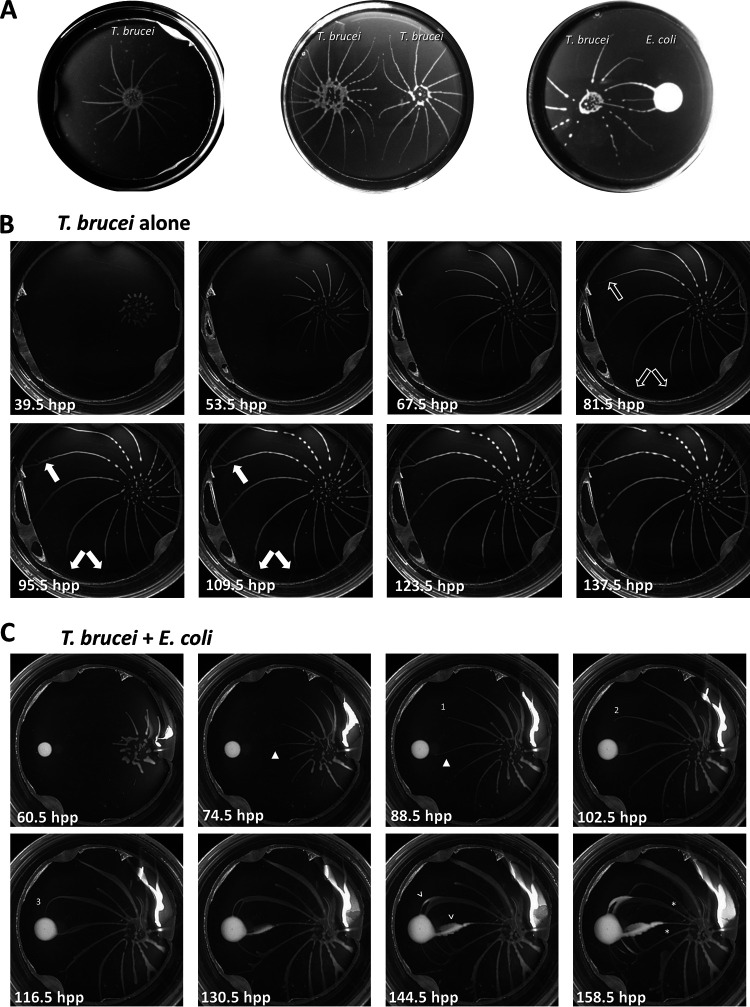
Socially behaving T. brucei is attracted to E. coli. (A) T. brucei on a semisolid surface engages in social motility (SoMo) (left). Projections of two groups of T. brucei originating from the same suspension culture are repelled by one another (center). T. brucei is attracted to E. coli (right). (B) Stills from a time-lapse video of T. brucei engaging in SoMo (Movie S1). Unfilled arrows point to projections before branching. Filled arrows point to projections that have formed branches. (C) Stills from a time-lapse video of T. brucei exhibiting positive chemotaxis toward E. coli (Movie S2). Time stamps are indicated in hours postplating (hpp). Numbers 1 to 3 indicate a projection that alters its path in response to E. coli. Closed arrowheads point to a change in curvature of the projection as it changes its path. Open arrowheads point to locations where E. coli has entered the projections. Asterisks indicate regions where a projection has crossed a different projection.

During social motility (SoMo), T. brucei cells engage in collective motility to form radial projections that have a clockwise curvature (when viewed from above; Fig. 1A, left) (19). When parasites in these projections sense other T. brucei cells, they actively avoid one another, either by stopping their forward movement or by changing their direction of movement (Fig. 1A, center) (19). We found, however, that when encountering E. coli on the SoMo plate, the parasite projections continue moving to make contact with the bacteria and even appear to alter their movement to move directly toward the bacteria (Fig. 1A, right).

10.1128/mSphere.00685-20.5 MOVIE S1 Time-lapse video of T. brucei engaging in SoMo. Still images were taken every 30 min for 162 h beginning 24 h postplating. Still images play back at 10 frames per second. Download Movie S1, AVI file, 16.0 MB. Copyright © 2020 DeMarco et al. 2020 DeMarco et al. This content is distributed under the terms of the Creative Commons Attribution 4.0 International license.

10.1128/mSphere.00685-20.6 MOVIE S2 Time-lapse video of T. brucei engaging in positive chemotaxis toward E. coli. Still images were taken every 30 min for 185 h beginning 24 h postplating using a Brinno TLC200 Pro time-lapse camera. Still images play back at 10 frames per second. Download Movie S2, AVI file, 19.0 MB. Copyright © 2020 DeMarco et al. 2020 DeMarco et al. This content is distributed under the terms of the Creative Commons Attribution 4.0 International license.

We hypothesized that the movement toward bacteria is chemotactic in nature, but we also considered whether it might instead reflect preferential growth in the direction of bacteria. To distinguish between these possibilities, we used time-lapse imaging to examine the dynamics of T. brucei movement. SoMo assays were performed with or without bacteria. Images were taken every 30 min over the course of 162 h (Fig. 1B, Movie S1) or 185 h (Fig. 1C, Movie S2) and compiled into movies. Plates were turned upside-down to prevent condensation on the lid from interfering with the analysis and then imaged from the bottom side of the plate. Therefore, the curvature of the projections observed in videos and time-lapse images is counterclockwise.

In the absence of bacteria, parasites moved continually outward, forming arced projections that radiated away from the inoculation site and rarely altered their general direction of movement (Fig. 1B and Movie S1). At early stages, projections maintained relatively even spacing and uniform width, having a single leading edge without branching. As projections neared the periphery, the space between projections increased, and parasites advanced from the lateral edge to form branches (Fig. 1B, arrows; Movies S1 and S3). The observation that branching only occurred when spacing between neighboring projections increased supports the idea (19) that inhibitory signals from parasites in adjacent projections prevent parasite movement from the side of projections. In some cases, thickening of a projection was observed prior to branching (Fig. S1, Movie S3), suggesting that cell density-dependent signals driving parasite movement outward (20) may overcome inhibitory signals between projections. Parasites in branches continued to adjust their movements so that they did not make contact with adjacent parasites (Fig. 1B, Fig. S1, Movies S1 and S3), demonstrating that parasite-dependent inhibitory signals were still active.

10.1128/mSphere.00685-20.1 FIG S1 T. brucei projections thicken prior to branching. Stills from a time-lapse video of T. brucei engaging in SoMo (Movie S3). The arrow points to the projection that thickens before branching. Time stamps are indicated in hours postplating (hpp). Download FIG S1, TIF file, 0.8 MB. Copyright © 2020 DeMarco et al. 2020 DeMarco et al. This content is distributed under the terms of the Creative Commons Attribution 4.0 International license.

10.1128/mSphere.00685-20.7 MOVIE S3 Time-lapse video of T. brucei engaging in SoMo. Still images were taken every 30 min for 161.5 h beginning 24 h postplating using a Brinno TLC200 Pro time-lapse camera. Still images play back at 10 frames per second. Download Movie S3, AVI file, 14.2 MB. Copyright © 2020 DeMarco et al. 2020 DeMarco et al. This content is distributed under the terms of the Creative Commons Attribution 4.0 International license.

Time-lapse imaging of SoMo assays carried out in the presence of a neighboring bacterial colony allowed us to define the point at which parasites sense and respond to bacteria, both spatially and temporally, and this revealed several important findings (Movies S2 and S4). First, these analyses clearly demonstrate that parasite movement toward bacteria is an active response and not simply an absence of avoidance. Notice, for example, that rather than continuing to the periphery of the plate, as occurs in the absence of bacteria, parasite projections curve sharply to move directly toward bacteria (Fig. 1C, numbers 1 to 3, 88.5 to 116.5 h postplating [hpp]). Moreover, in the presence of bacteria, projections change curvature from counterclockwise to clockwise (Fig. 1C, closed arrowheads, 74.5 and 88.5 hpp) and exhibit extensive branching (Fig. 1C, 130.5 hpp to 158.5 hpp), with branches moving directly toward the bacteria. Second, these changes in movement occur at a large distance from the edge of the bacterial colony, indicating that parasites are responding to a chemical cue derived from the bacteria, rather than detecting the bacteria by direct contact (Fig. 1C, Movies S2 and S4).

10.1128/mSphere.00685-20.8 MOVIE S4 Time-lapse video of T. brucei engaging in positive chemotaxis toward E. coli. Still images were taken every 30 min for 183.5 h beginning 24 h postplating using a Brinno TLC200 Pro time-lapse camera. Still images play back at 10 frames per second. Download Movie S4, AVI file, 17.8 MB. Copyright © 2020 DeMarco et al. 2020 DeMarco et al. This content is distributed under the terms of the Creative Commons Attribution 4.0 International license.

A third important result to come from time-lapse studies is that the timescale of the response rules out the possibility that movement toward bacteria simply represents preferential or faster growth in this direction. For example, between 88.5 and 116.5 hpp (Fig. 1C, numbers 1 to 3), parasites turned sharply toward bacteria and advanced to contact the bacterial colony. This projection impacted the bacteria at 109 hpp (Movie S2). Growing on plates, T. brucei has a doubling time of approximately 24 h (19); thus, the 20.5-h time interval between 88.5 hpp and 109 hpp represents slightly less than one cell doubling time, yet the parasites moved 22.6 mm (Movie S2), which corresponds to approximately 1,046 cell lengths (26). Clearly, movement over this distance is too fast to be accounted for by only cell doubling. Therefore, parasites alter their movement to move directly toward bacteria, in response to a signal that acts at a distance.

In the moments before parasite projections impact the bacterial colony, we see individual parasites move directly from parasite projections into the bacterial colony (Movies S5 and S6). After contact, parasites spread out as they infiltrate the bacterial colony (Movies S5 and S6). Meanwhile, bacteria from the colony advance outward along parasite projections (Fig. 1C, open arrowheads). As bacteria advance along one projection, parasites from adjacent projections become attracted to the position now occupied by bacteria (Fig. 1C, 144.5 and 158.5 hpp). Therefore, repositioning of the bacterial population directly correlates with a change in position of the attractant source. As parasites move to this attractant, they now even cross other projections of parasites to reach the bacteria (Fig. 1C, asterisks, 158.5 hpp), a phenomenon never observed in the absence of attractant. This indicates that the attractive cue from the bacteria is stronger than the repulsive cue that otherwise prevents contact and crossing of projections (19). Altogether, time-lapse video analysis demonstrated that parasites in projections are not exhibiting preferential growth toward E. coli but are actively directing their movement toward it through positive chemotaxis in response to an attractant that acts at a large distance from the source.

10.1128/mSphere.00685-20.9 MOVIE S5 Live video of T. brucei cells at the tip of a projection upon initial impact with an E. coli colony. Cells were imaged at ×20 magnification, and video was recorded and played back at 30 frames per second. Download Movie S5, AVI file, 18.6 MB. Copyright © 2020 DeMarco et al. 2020 DeMarco et al. This content is distributed under the terms of the Creative Commons Attribution 4.0 International license.

### The attractant is diffusible and requires actively growing E. coli.

**FIG 2:**
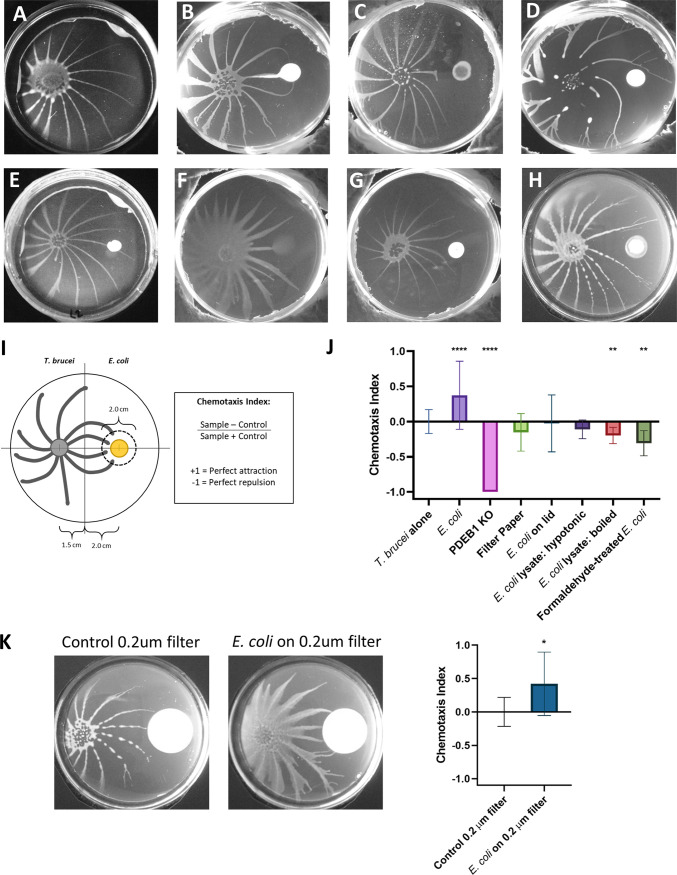
The attractant is diffusible and requires actively growing E. coli. (A to H) Representative pictures of each condition tested in the chemotaxis assay. T. brucei alone (A), E. coli (B), PDEB1 KO (C), filter paper (D), E. coli on lid (E), E. coli lysate, hypotonic (F), E. coli lysate, boiled (G), formaldehyde-treated E. coli (H). (I) Requirements for attraction were quantified by a chemotaxis index, defined as the number of projections entering the 2-cm-diameter red circle in the experimental sample subtracted by the number of projections entering the same circle on control plates (T. brucei alone) divided by the total number of projections in both samples. A schematic of the chemotaxis assay is shown. (J) Each condition and its chemotaxis index are shown. Error bars represent the standard error of the mean (SEM). Unpaired two-tailed *t* test with Welch’s correction was used to measure significance compared to the T. brucei-alone control: ****, *P* < 0.0001; **, *P* < 0.01. (K) A chemotaxis index was calculated for T. brucei in response to E. coli growing on a 0.2-μm filter compared to a 0.2-μm filter alone. Representative images are shown. Error bars represent the SEM. An unpaired two-tailed *t* test with Welch’s correction was used to measure significance compared to the T. brucei in response to the 0.2-μm filter-alone control; *, *P* < 0.05.

To define characteristics of the attractant, we employed a quantitative chemotaxis assay (Fig. 2I) developed based on similar assays used to study chemotaxis in parasitic worms (27). In this assay a chemotactic index is calculated for each sample by determining the number of projections that enter a 2-cm diameter centered around the sample compared to how many projections enter a circle centered at the same position when no sample is present. A positive chemotaxis index indicates attraction, while a negative index indicates repulsion, and “perfect” attraction and repulsion are defined as +1 and –1, respectively. In this assay, a colony of live bacteria has a positive chemotactic index, +0.37, indicating that T. brucei is strongly attracted to E. coli (Fig. 2B and J). We previously showed that T. brucei is repelled by other groups of T. brucei (19). Therefore, as a negative control, we used a T. brucei PDEB1 knockout (KO) mutant that does not form projections (8) and produces a colony approximately equal in size to bacterial colonies grown on SoMo plates (Fig. 2C). We found that T. brucei was “perfectly” repelled by PDEB1 KO T. brucei, with a chemotaxis index of –1 (Fig. 2J), supporting the capacity of the assay to distinguish attractive versus repulsive chemotaxis.

We next considered whether attraction to bacterial colonies might simply reflect a response to a physical perturbation in the agarose surface created by the physical presence of the bacterial colony. To assess this, the chemotactic index of a piece of filter paper was tested, and T. brucei showed no significant chemotactic response (Fig. 2D and J), reinforcing the hypothesis that the response to bacteria is a chemotactic response.

Bacteria can produce both volatile—released into the air—and soluble compounds, which can serve as chemotactic cues (28, 29). To differentiate between these, we first asked whether T. brucei would respond positively to aerosolized volatile compounds. To do this, we employed a variation in the chemotaxis assay in which E. coli was plated on the lid of the petri dish while T. brucei was inoculated on the bottom. T. brucei showed no response to E. coli on the lid (Fig. 2E and J), indicating that the attractive cue is nonvolatile and suggesting that it is a soluble factor that diffuses through the culture medium.

To test if the attractant was diffusible through the culture medium, we assessed chemotaxis to E. coli plated on 0.2-μm filter discs placed on the SoMo plate. The filter disc prevents bacteria from directly contacting the culture medium but allows small molecules to diffuse through it. In this case, the chemotactic index was determined relative to a 0.2-μm filter disc with no bacteria. T. brucei were attracted to E. coli grown on the 0.2-μm filter, with a positive chemotactic index of +0.42 (Fig. 2K), indicating that attraction occurs in response to a factor smaller than 0.2 μm that diffuses through the culture medium. It is important to note that the attractant may be a signal produced directly from the bacteria, or it may be a product of a chemical reaction between a factor produced by the bacteria and a substance present in the culture medium.

Because T. brucei is attracted to a diffusible cue emanating from E. coli, we next asked if this required dead or dying bacteria. When bacteria die, they often lyse, releasing intracellular metabolites into the environment, which can serve as nutrient sources for other microbes (30, 31), so we asked if T. brucei was attracted to products released from lysed E. coli. To determine the number of lysed bacterial cell equivalents to test, we determined the number of E. coli cells present at 96 hpp (Fig. S2) because parasites show an attractive response to bacteria within 96 hpp (Fig. 1C). First, hypotonically lysed bacteria were tested to determine if the attractant might be a protein released from lysed bacteria, but no chemotactic effect was seen (Fig. 2F and J). Second, boiled E. coli cell lysates were tested (Fig. 2G). Boiling E. coli would denature proteins and inactivate heat labile compounds, but other potential metabolites would still be present; however, boiled lysates were repulsive to T. brucei (Fig. 2J). We also assessed the chemotactic index of dead but nonlysed E. coli, using formaldehyde-killed bacteria, and found that T. brucei was repelled by formaldehyde-killed bacteria (Fig. 2H and J). Finally, conditioned medium from liquid-cultured E. coli was concentrated and assayed, but no attraction or repulsion was seen (data not shown). Taken together, these results indicate that the attractant is diffusible through the culture medium, and actively growing bacteria are required for its production. Efforts to isolate or identify the attractant have so far been unsuccessful.

10.1128/mSphere.00685-20.2 FIG S2 Growth of E. coli colonies on SoMo plates and the curvature of the tip of T. brucei projections increases in the presence and absence of E. coli. (A) Growth of E. coli colonies was measured on SM-supplemented agarose plates in the absence of antibiotics over the course of 4 days. (B) The angle of curvature of the tip of the projections in panels A and B of Fig. 5 are plotted over time (hours postplating). (C) A schematic of how the angle of curvature was determined in panel B is shown. Download FIG S2, TIF file, 0.2 MB. Copyright © 2020 DeMarco et al. 2020 DeMarco et al. This content is distributed under the terms of the Creative Commons Attribution 4.0 International license.

### Projections of parasites accelerate upon sensation of an attractant.

**FIG 3:**
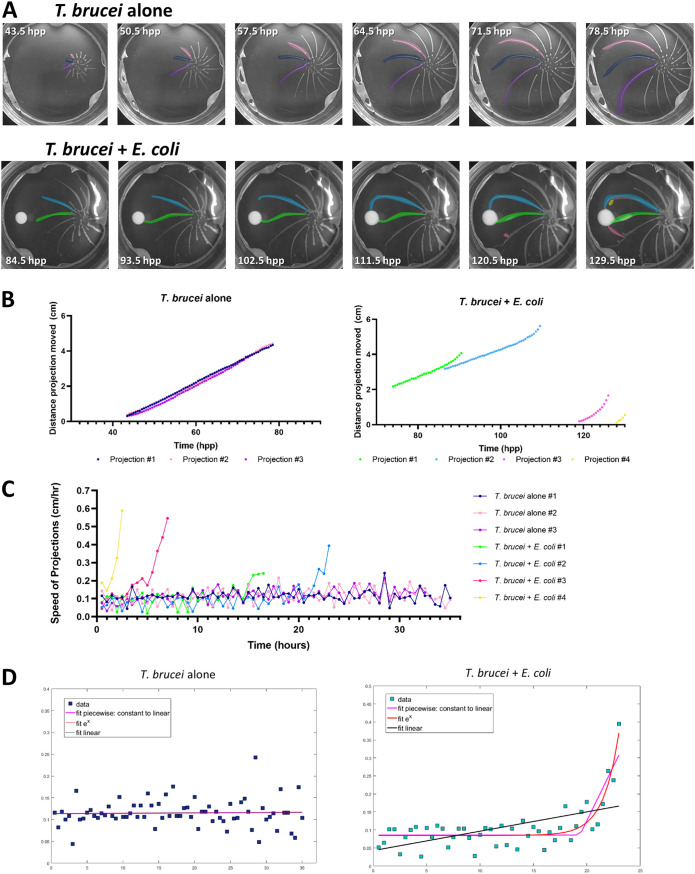
Projections of parasites accelerate upon sensation of attractant. (A) Representative images of T. brucei engaging in SoMo alone (upper) (Movie S1) or with E. coli present (lower) (Movie S2). Projections are pseudocolored and match the plot colors shown in panel B. Time stamps are indicated in hours postplating (hpp). (B) The distance each projection moved was measured over time from the time-lapse videos shown in panel A. (C) The speed of each projection is plotted over time with the colors of each projection corresponding to their respective colors shown in panels A and B. (D) Nonlinear regression models designed in MATLAB were used to model the best fit of the speed versus time data for projections in the presence of E. coli. For T. brucei alone, projection 1 is shown, and for T. brucei plus E. coli, projection 2 is shown. The graphs with the regression models for the other projections can be found in Fig. S3F and G. The pink line represents the piecewise function, the red line is for an exponential function, and the black line represents a linear function. The colors of the data points correspond to the respective pseudocolors for each projection.

The attraction of social T. brucei to E. coli presents an opportunity to investigate changes in T. brucei cell behavior underlying chemotaxis. In time-lapse video analysis, we noticed that projections appeared to speed up just before they made contact with the bacterial colony (Fig. 3A, Movies S1 and S2). To quantify this, we measured the distance each projection travelled between each frame of the time-lapse video, in either the presence or absence of bacteria. A plot of distance traveled over time was then generated for each projection (Fig. 3B).

10.1128/mSphere.00685-20.3 FIG S3 Velocity analysis of projections from additional time-lapse videos (Movies S3 and S4) in both the presence and absence of bacteria. (A) Representative images of T. brucei engaging in SoMo alone (upper) (Movie S3) or T. brucei engaging in SoMo with E. coli present (lower) (Movie S4). Projections are pseudocolored and match the colors shown in panel B. Time stamps are indicated in hours postplating (hpp). (B) The distance each projection moved was measured over time from the time-lapse videos shown in panel A. (C) The speed of each projection is plotted over time, with the colors of each projection corresponding to their respective colors shown in panels A and B. (D and E) Nonlinear regression models designed in MATLAB were used to model the best fit of the speed versus time data for projections in both the absence (D) and presence (E) of E. coli. The pink line represents the piecewise function, the red line is for an exponential function, and the black line represents a linear function. The colors of the data points correspond to the respective pseudocolors for each projection. (F and G) Nonlinear regression models designed in MATLAB were used to model the best fit of the speed versus time data for projections in both the absence (F) and presence (G) of E. coli for the projections shown in Fig. 3. The pink line represents the piecewise function, the red line is for an exponential function, and the black line represents a linear function. The colors of the data points correspond to the respective pseudocolors for each projection. Download FIG S3, TIF file, 0.8 MB. Copyright © 2020 DeMarco et al. 2020 DeMarco et al. This content is distributed under the terms of the Creative Commons Attribution 4.0 International license.

In the absence of bacteria, projections moved with mostly constant speed, as indicated by the constant slope of the line generated by the distance versus time analysis (Fig. 3B, left). Distance versus time measurements were fit to a linear or quadratic regression model (Table 1). All three projections fit the linear model very well (R^2^ = 0.99). Although the quadratic model also fit (R^2^ = 0.99), the constant in front of the *x*^2^ value in each of the three equations was always very small, indicating that in the absence of bacteria, the projections do, in fact, move with a constant speed (∼0.1 cm/h) (Table 1, Table S1). For projections moving in the presence of bacteria, the distance traveled versus time analysis indicated that the projections moved at a constant speed at first, as indicated by the constant slope at early time points. In the hours before impact with the bacteria, however, the slope of the line continually increased, indicating that the projections of parasites were accelerating toward the bacteria (Fig. 3B, right).

**TABLE 1:** Equations of the regression models for T. brucei projections in the absence of bacteria—distance versus time^*a*^

Projection	Linear regression (*y* = *ax* + *b*)	Quadratic regression (*y* = *ax*^2^ + *bx* + *c*)
T. brucei alone 1	*y* = 0.1172*x* − 4.8248 R² = 0.9996	*y* = −2e-05*x*^2^ + 0.1191*x* − 4.8819 R² = 0.9996
T. brucei alone 2	*y* = 0.1184*x* − 4.9054 R² = 0.9975	*y* = 0.0006*x*^2^ + 0.0441*x* − 2.7047 R² = 0.9997
T. brucei alone 3	*y* = 0.1209*x* − 5.1828 R² = 0.9965	*y* = 0.0008*x*^2^ + 0.027*x* − 2.4479 R² = 0.9995

a Equations for the best fit for both a linear regression and quadratic regression model were calculated for the indicated T. brucei projection in the absence of bacteria shown in Fig. 3B. R^2^ values were calculated in Microsoft Excel for each regression analysis.

10.1128/mSphere.00685-20.4 TABLE S1 Equations of the regression models for T. brucei projections from Movie S3 (distance versus time) and for Movies S3 and S4 (speed versus time). Equations for the best fit for both a linear regression and a quadratic regression model were calculated for the indicated T. brucei projection in the absence of bacteria shown in Fig. S3B. R^2^ values were calculated in Microsoft Excel for each regression analysis. Additionally, equations for the best fit for a nonlinear fitting algorithm for a piecewise, exponential, and linear function were calculated for the speed versus time data of T. brucei projections in the presence and absence of E. coli. R^2^ values were calculated using the nonlinear fit model in MATLAB for each regression analysis. Download Table S1, DOCX file, 0.01 MB. Copyright © 2020 DeMarco et al. 2020 DeMarco et al. This content is distributed under the terms of the Creative Commons Attribution 4.0 International license.

Because it is difficult to fit an equation to a line that changes from a constant slope to an increasing slope, we plotted the change in distance between each time point (i.e., the derivative), which represents the speed versus time (Fig. 3C). While there was noise in the speed versus time analysis for each projection measured, all projections in the presence of bacteria clearly increased their speed above the baseline speed of projections in the absence of bacteria (Fig. 3C, Fig. S3). For example, in the final 30 min before they impacted bacteria, projections reached speeds of 0.24 to 0.59 cm/h (Fig. 3C).

To model the speed change of projections in the presence of bacteria, we used different nonlinear regression models designed in MATLAB to analyze the speed versus time data (Fig. 3D, Table 2). Because projections appeared to move with a constant speed early and then accelerate before colliding with the bacteria, we first asked how well the data could be modeled by a piecewise function that began with a line with zero slope (i.e., constant speed) and then at an unknown time point (denoted by the term *k*) changed to a line with a constant and positive slope (i.e., increasing speed) (Table 2). We also asked how well the speed data fit an exponential equation, which would give an equation with an almost zero slope at early time points and then continuously change to an increasing slope. Finally, we also asked how well the data fit a linear equation, which would give an equation of a line with an unchanging slope. We found that while the piecewise function that modeled a zero slope to a constant slope fit the speed data well (R^2^ = 0.522 to 0.929), the exponential regression provided a slightly better model for the data (R^2^ = 0.544 to 0.973) (Table 2). Linear regression did not fit the data as well as the other two models (Table 2, Table S1). These same models were applied to the speed of projections in the absence of bacteria, and most models gave equations of lines with close to zero slope, confirming that these projections move with a constant speed. These analyses indicate that in the presence of bacteria, projections move with a mostly constant speed, but in the hours before they reach the bacteria, the group increases its speed dramatically. Analyses of additional time-lapse videos in the presence or absence of bacteria are consistent with these findings (Fig. S3, Table S1).

**TABLE 2:** Equations of the regression models for T. brucei projections in the absence or presence of bacteria—speed versus time^*a*^

Projection	Piecewise function: constant to a line (for *x* ≤ *k*, *y* = *a*; for *x* > *k*, *y* = *bx* + *c*)	Exponential regression (*y* = *a e^bx^* + *c*)	Linear regression (*y* = *ax* + *b*)
T. brucei alone 1	For *x* ≤ 0, *y* = 0.1 For *x* > 0, *y* = 8.95e^−5^ *x* + 0.11 R^2^ = −0.0138	*y* = 2.47 e^3.6e-5^ *^x^* − 2.35 R^2^ = −0.0138	*y* = 8.95e^−5^ *x* + 0.11 R^2^ = −0.0138
T. brucei alone 2	For *x* ≤ 0, *y* = 0.09 For *x* > 0, *y* = 8.7e^−4^ *x* + 0.099 R^2^ = 0.0308	*y* = 11.2 e^7.74e−5^ *^x^* − 11.1 R^2^ = 0.0308	*y* = 8.70e^−4^ *x* +0.01 R^2^ = 0.0308
T. brucei alone 3	For *x* ≤ 0, *y* = 0.076 For *x* > 0, *y* = 0.00199*x* + 0.085 R^2^ = 0.278	*y* = 9.224 e^2.15e−4^ *x* − 9.14 R^2^ = 0.278	*y* = 0.00199*x* + 0.085 R^2^ = 0.278
T. brucei plus E. coli 1	For *x* ≤ 12.21, *y* = 0.094 For *x* > 12.21, *y* = 0.033*x* − 0.309 R^2^ = 0.522	*y* = 8.67e^−5^ e^0.458^ *^x^* + 0.090 R^2^ = 0.544	*y* = 0.0065*x* + 0.0596 R^2^ = 0.304
T. brucei plus E. coli 2	For *x* ≤ 19.34, *y* = 0.086 For *x* > 19.34, *y* = 0.061*x* − 1.088 R^2^ = 0.674	*y* = 3.87e^−8^ e^0.687^ *^x^* + 0.085 R^2^ = 0.754	*y* = 0.0054*x* + 0.0428 R^2^ = 0.3
T. brucei plus E. coli 3	For *x* ≤ 3.40, *y* = 0.1 For *x* > 3.40, *y* = 0.104*x* − 0.254 R^2^ = 0.874	*y* = 8.66e^−3^ e^0.571^ *^x^* + 0.078 R^2^ = 0.957	*y* = 0.0618*x* − 0.0212 R^2^ = 0.784
T. brucei plus E. coli 4	For *x* ≤ 1.45, *y* = 0.167 For *x* > 1.45, *y* = 0.376*x* − 0.377 R^2^ = 0.929	*y* = 3.17e^−3^ e^1.97^ *^x^* + 0.154 R^2^ = 0.973	*y* = 0.196*x* − 0.002 R^2^ = 0.673

a Equations for the best fit for a nonlinear fitting algorithm for a piecewise, exponential, and linear function were calculated for the speed versus time data of T. brucei projections in the absence or presence of E. coli. R^2^ values were calculated using the nonlinear fit model in MATLAB for each regression analysis.

### Individual cell motility within the group is constrained when sensing an attractant.

**FIG 4:**
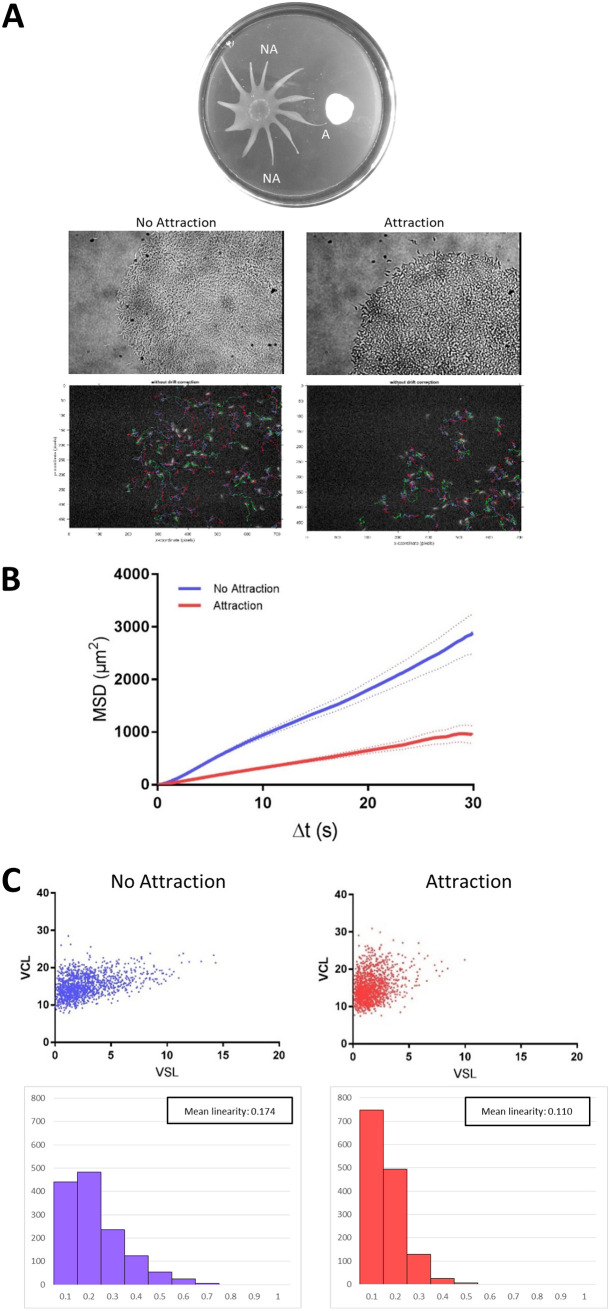
Individual cell motility within the group becomes more constrained in the presence of attractant. (A) A representative SoMo plate is shown with a projection undergoing chemotaxis to E. coli (attraction, A) and two projections not engaged in chemotaxis (no attraction, NA). Representative phase contrast images of the tips of projections at ×20 magnification are shown. Fluorescent images of the same tips of projections show GFP-tagged cells superimposed with their cell traces over a 30-s time frame. (B) Mean-squared displacement of individual cells at the tips of projections undergoing chemotaxis (attraction) or not (no attraction). The data acquired were from 37 videos with 1,368 total tracks (no attraction) or 22 videos with 1,403 total tracks (attraction). (C) Curvilinear versus straight-line velocity plots with corresponding linearity plots are shown for attraction and no-attraction conditions.

To assess changes in individual cell behavior occurring in response to the attractant, untagged wild-type cells were mixed with 10% green fluorescent protein (GFP)-expressing cells. Through the use of a cell-tracking algorithm (32), the movements of individual GFP-tagged cells were assessed within the group. Individual cells at the tips of projections that were either not attracted to E. coli (*n* = 1,368 cell tracks) or attracted to E. coli (*n* = 1,403 cell tracks) were traced in 30-s videos (Fig. 4A). We assessed mean-squared displacement (MSD), which tracks the *x*/*y* displacement during specific time intervals, thus taking into account both the speed of cells and how far they move from their initial locations. We found that cells undergoing chemotaxis to E. coli had a lower MSD than cells in projections that were not undergoing chemotaxis (Fig. 4B). The lower MSD could mean that individual cells move more slowly in response to the attractant or that they alter how they move.

To examine this further, we plotted the distribution of each cell’s curvilinear velocity versus straight-line velocity. Straight-line velocity represents the cell’s displacement over time—basically, the average speed of the cell as it travelled from its starting point to its final position, regardless of the path it took. Curvilinear velocity, however, takes into account the total distance of the path that the cell travelled in the same amount of time. We found that cells sensing an attractant had reduced straight-line velocity compared to cells not engaged in chemotaxis, suggesting that when an attractant is detected, parasites restrict their motion to smaller and more curving paths to remain near the attractant (Fig. 4C). A similar behavioral switch in which “random walk” behavior of individual cells becomes more “constrained” has been described for bacterial chemotaxis to K^+^ ions within a biofilm (33). To quantify this change in T. brucei, we calculated the linearity for each cell by taking the ratio of straight-line velocity to curvilinear velocity. The mean linearity for cells not undergoing chemotaxis was 0.174, while the mean linearity for those undergoing chemotaxis was significantly decreased at 0.110 (two-tailed *t* test, *P* < 0.0001).

### Group movement and single-cell movement are correlated.

**FIG 5:**
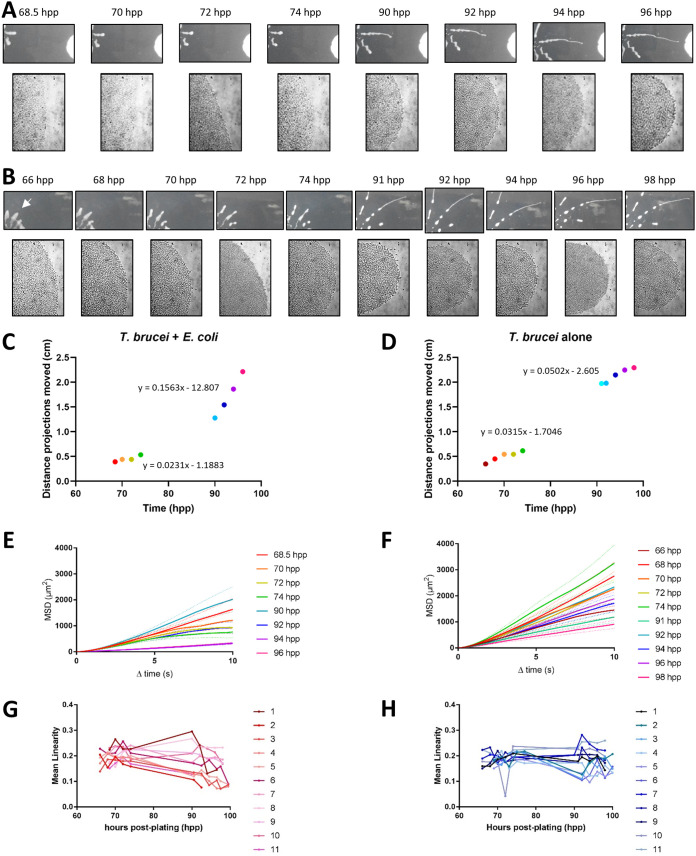
Changes in group movement correlate with changes in single cell movement during positive chemotaxis. The speed of projections and MSD of individual cells within tips of these projections were examined over time in samples with or without E. coli. (A) Sequential images of a projection moving toward E. coli. At each time point, the tip of the projection is shown at ×20 magnification (lower row). (B) Projection of T. brucei alone analyzed as described in panel A. (C and D) The distance from the point of origin to the projection tip is plotted as a function of time for T. brucei projections moving in the presence (C) or absence (D) of E. coli. Equations for line of best fit using a linear regression are shown above the corresponding portions of each graph. (E and F) Mean-squared displacement was determined for individual cells in the tip of each projection shown in panels A and B. The line colors correspond to the colors used for time points in panels C and D. (G and H) The mean linearity of individual cells at the tips of projections was plotted over time for 11 projections each of T. brucei in the presence (G) and absence (H) of E. coli.

To determine if acceleration of projections toward E. coli correlates directly with constrained motility of individual cells, we monitored projection movement and individual cell motility in parallel as a function of time. SoMo assays were done with a mixture of 1% GFP-tagged cells mixed with untagged cells. We took 30-s movies of individual fluorescent cells at the tips of the projections at the same time points as photographs of projections over a 2-day time course. In a representative SoMo plate of T. brucei coinoculated with E. coli, the distance projections moved was measured every 2 h from 68.5 to 74 h postplating and from 90 to 96 h postplating, moments before the projection collided with the bacteria (Fig. 5A). As expected, projections increased their speed as they moved toward bacteria, accelerating from 0.0231 cm/h to 0.1563 cm/h (Fig. 5C, Table 3). In contrast, in the absence of bacteria, the speed of the projection did not change substantially—0.0315 cm/h to 0.0502 cm/h (Fig. 5D, Table 3). It should be noted that these speeds were slower overall than those seen in the time-lapse videos in Fig. 3, which may be due to slight technical differences between the two assays. Over the time course analysis, the projection tips became more curved (Fig. 5A and B), but this was observed regardless of whether bacteria were present (Fig. S2B), indicating that it is likely a characteristic of advancing projections.

**TABLE 3:** Equations for the lines of best fit for T. brucei projections in the time course analyses—distance versus time^*a*^

Organism(s)	Equations	
E. coli plus T. brucei	68.5–74 hpp	90–96 hpp
	Linear regression	Linear regression
	*y* = 0.0231*x* − 1.1883 R² = 0.8609	*y* = 0.1563*x* − 12.807 R² = 0.9959
*T. brucei* alone	66–74 hpp	91–98 hpp
	Linear regression	Linear regression
	*y* = 0.0315*x* − 1.7046 R^2^ = 0.9245	*y* = 0.0502*x* − 2.605 R^2^ = 0.9572

a Equations for the line of best fit for each section of the graphs in Fig. 5C and D were determined using a linear regression analysis. R^2^ values were calculated in Microsoft Excel for each regression analysis.

To determine how the movement of individual cells within the projections changed over time, we monitored their movements in the same projections used for the speed analysis described above. In the presence of E. coli, parasite cells exhibited increasingly constrained motility over time (Fig. 5E), consistent with the results from Fig. 4B and C. While there was variation in the MSD of individual cells over time, a significant decrease in MSD was clear by 94 and 96 hpp (one-way analysis of variance [ANOVA], *P* < 0.0001). Interestingly, the decrease in MSD seen in the presence of bacteria was not observed until the last two time points before the projection impacted the bacteria (Fig. 5E, 94 and 96 hpp), even though the projection as a whole had already begun increasing its speed in the prior two time points (Fig. 5C, 90 and 92 hpp). This finding suggests that while changes in group and individual movement in the presence of bacteria are correlated, projection speed increases first, and individual cells constrain their motility later. We suspect there is some aspect of individual cell behavior that leads to the increased speed of the group as a whole, but so far, this has not been revealed in our current assays. In the absence of bacteria, T. brucei cells showed variation in their constraint of motion over time (Fig. 5F). However, no single time point exhibited a significant decrease in MSD from all of the others (one-way ANOVA) (Fig. 5F), indicating that cells in the absence of bacteria were not changing how they moved within the projection, in contrast to how individual cells behave in the presence of bacteria.

When individual cells in multiple projections were monitored over time, this same trend held true (Fig. 5G and H). In the presence of bacteria, the mean linearity of cells in projections decreased over time (Fig. 5G), indicating that their movements were becoming more constrained as they moved toward the bacteria. In the absence of bacteria, the mean linearity was variable, and although some decrease occurred, the large drop seen during the final time points in the presence of bacteria was not observed (Fig. 5H). Taken together, these time course experiments indicate that in response to bacteria, the speed of projections increases, and subsequently, individual cells within projections constrain their motion.

## DISCUSSION

We have shown that socially behaving T. brucei engage in positive chemotaxis toward a diffusible cue produced by actively growing E. coli, and we have begun to elucidate the changes in group and individual cell behaviors that characterize this response. While chemotaxis has been well studied in bacteria and other protozoans (1–3), to our knowledge, our results provide the first demonstration of positive chemotaxis in T. brucei and illustrate the capacity for interkingdom interactions between bacteria and protozoa.

In its tsetse fly host, T. brucei must undergo a series of specific migrations and differentiations as it moves from the fly midgut to the proventriculus and, finally, to the salivary glands. It is therefore reasonable to anticipate that T. brucei may employ chemotaxis to direct movement in response to signals in specific fly tissues. Supporting this idea, prior work connected cAMP as a second messenger to control of parasite group movements *in vitro* (23, 24, 34), and recent work demonstrated that T. brucei requires an intact cAMP signaling pathway in order to progress through tsetse fly tissues *in vivo* (8). The idea that chemotactic cues direct parasite progression through their insect vectors has been proposed for other kinetoplastid parasites, including *Leishmania* spp. and Trypanosoma cruzi, which have been shown to engage in chemotaxis *in vitro* (9, 11, 12). Our discovery of positive chemotaxis in T. brucei demonstrates that, in addition to moving away from external signals (19), these organisms can detect and move toward specific signals in their extracellular environment.

The ability to sense and respond to signals is also expected to be important for T. brucei within its mammalian hosts. Prior work has shown that in addition to the bloodstream and central nervous system, skin and adipose tissues represent important reservoirs contributing to pathogenesis and transmission (15–17). Mechanisms underlying T. brucei tropism to extravascular tissues remain to be determined, but positive chemotaxis could be involved. A study of the malaria parasite Plasmodium berghei in a mammalian host found that as parasites moved closer to blood vessels, their trajectories became more constrained (35), which mirrors the more constrained individual cell motility exhibited by T. brucei in response to attractant (Fig. 4C, 5A, E, and G). Chemotaxis may also help T. brucei evade the immune system, analogous to what has been proposed for evasion of host neutrophils by *Leishmania* parasites (10). Motility is essential for T. brucei virulence in the mammalian host, perhaps allowing for quick changes in direction to avoid immune cells (32). Thus, the change in individual cell motility observed as parasites move toward an attractant (Fig. 4C, 5A, E, and G) may be an important mechanism to infiltrate tissues and/or evade immune cells within the host.

The question arises as to why T. brucei exhibits chemotaxis toward bacteria. Notably, although E. coli is not typically present within the tsetse fly, the fly is home to three species of endosymbiotic Gram-negative bacteria, Sodalis glossinidius, Wigglesworthia glossinidia, and *Wolbachia* spp. (36). While *Wolbachia* is restricted to the reproductive tract, *Wigglesworthia*, an obligate endosymbiont, is found intracellularly in bacteriocytes within a specialized structure called the bacteriome, near the anterior midgut, and extracellularly in the milk glands (37, 38). *Sodalis* is found throughout the fly midgut and in a variety of other tissues (36). Evidence for functional interactions between T. brucei and bacterial endosymbionts of the tsetse comes from work demonstrating T. brucei’s reliance on bacterial products during fly transmission (36). For example, *Wigglesworthia* produces folate and phenylalanine, but T. brucei cannot. T. brucei does, however, encode transporters for these metabolites (39). Similarly, T. brucei encodes an incomplete threonine biosynthesis pathway (40). While the tsetse cannot provide homoserine that is necessary for T. brucei threonine biosynthesis (41), *Sodalis* can (42). A long-standing interaction between T. brucei and *Sodalis* is also evidenced by an example of horizontal gene transfer of the gene phospholipase A1 from *Sodalis* to T. brucei (43, 44). Of note, numerous studies of lab-reared and field-caught tsetse flies have demonstrated that the presence of *Sodalis* in the tsetse increases the likelihood of infection by T. brucei (45–48). Therefore, T. brucei likely interacts closely with *Sodalis* during the transmission cycle, and chemotaxis toward *Sodalis* would be advantageous. While *Sodalis* can be cultured *in vitro*, we were unable to culture it on T. brucei medium, or vice versa, and thus the chemotactic index of T. brucei toward *Sodalis* could not be determined.

In addition to containing three endosymbiotic bacteria, the midgut of field-caught tsetse flies harbors a wide variety of bacterial species, with variation among different species of tsetse flies and geographic distribution (48). The most common genera of bacteria found in tsetse fly midguts included *Enterobacter*, *Enterococcus*, and Acinetobacter (48). While the three endosymbionts discussed above are all Gram-negative bacteria, other common genera found in the fly midgut encompass both Gram-negative and Gram-positive bacterial species. We did not detect attraction or repulsion toward the Gram-positive bacterium B. subtilis (data not shown). Future work will be needed to assess whether T. brucei shows chemotaxis to other examples of Gram-positive and Gram-negative bacteria.

The exact *in vivo* correlates of group movements observed in SoMo remain unclear. However, our findings, together with recent work (8, 20, 21, 23, 24, 49), clearly illustrate the value of SoMo for uncovering novel aspects of trypanosome biology that are relevant *in vivo*. The discovery here of BacSoMo, positive chemotaxis in T. brucei, and development of quantitative chemotaxis assays enabled us to begin investigating cellular mechanisms underlying directed movement in these pathogens. These systems enable studies at the scale of groups of cells and at the level of individual cells within groups. In bacteria, both scales of analysis have provided important insights. In *Pseudomonas* and *Myxococcus*, for example, analyses of group chemotaxis led to the discovery of signaling molecules and systems that regulate bacterial social behavior and motility on a surface (50, 51). In cyanobacteria, studies of individual cell movements within a group revealed how movement of individuals can dictate the trajectory of the group during the phototactic response (52). Thus, our analyses of changes in both group and individual cell behavior in response to attractant set the stage for further study of the cellular and molecular mechanisms underlying these responses.

Looking forward, the quantitative chemotaxis assays reported here will serve as an important tool to enable dissection of cellular and molecular mechanisms used by trypanosomes to detect and respond to environmental signals. As a straightforward *in vitro* assay, screens for small molecules that inhibit or alter trypanosome sensory behavior without affecting parasite fitness could identify novel transmission-blocking targets. Additionally, RNA interference (RNAi) library screens for genes involved in sensory, signaling, or other chemotactic functions could be identified, further elucidating signaling systems required for parasite transmission and pathogenesis. Overall, the identification of positive chemotaxis in T. brucei will lead to deeper insights into how parasites sense and respond to cues in their changing host environments, facilitating the development of novel therapeutic and transmission-blocking strategies.

## MATERIALS AND METHODS

### Trypanosomes.

T. brucei subsp. *brucei* 29-13 procyclic culture forms were used in this study (53). Parasites were cultured in SM medium with 10% heat-inactivated fetal bovine serum (FBS) at 28°C and 5% CO_2_. GFP-tagged 29-13 cells were generated by transfection with SpeI-linearized pG-eGFP-BLAST (gift of Isabel Roditi, University of Bern) as described previously (24). PDEB1 knockout cells were generated in the 29-13 background by two sequential rounds of homologous recombination using pTub plasmids conferring resistance to blasticidin and phleomycin (54). For regions of homology, 452 bp upstream and 635 bp downstream of the PDEB1 coding sequence were used, the same regions used to independently create a PDEB1 KO cell line as described in reference 8. Transfection of the knockout plasmids was performed as described previously (55). Primers used to amplify the PDEB1 regions of homology were described previously in reference 8 and are also listed here as follows: upstream forward (FWD), atatGCGGCCGCTGCATTATGTTACTTGGGGGCA; upstream reverse (REV), atatCTCGAGGACGTAGTGTCCAACTGTGC; downstream FWD, atatGGATCCAGTCAGTTGACCGGTGGTAG; downstream REV, atatTCTAGACCGCCACAACTCCCTCTTAC.

### Bacteria.

E. coli strain DH5α with an ampicillin resistance plasmid was used for all experiments. E. coli from a glycerol stock was grown overnight in SM. Then, 0.3 μl of log-phase E. coli (3 × 10^8^ to 5 × 10^8^ cells/ml) was inoculated on SoMo plates. Note that 100 μg/ml of carbenicillin was added to overnight cultures of E. coli, but no antibiotics were added to SoMo plates (SoMo assays were always done in the absence of antibiotics). Bacterial growth on SoMo plates was monitored for 4 days by washing colonies off independent SoMo plates with no antibiotics twice per day and measuring the optical density at 600 nm (OD_600_) for 3 biological replicates.

### Social motility assay.

Plates for social motility assays were prepared based on reference 19. Briefly, a solution of 4% (wt/vol) SeaPlaque GTG agarose (Lonza) in MilliQ water was sterilized for 30 min at 250°C. Water that evaporated was replaced with sterile MilliQ water after heating, and the solution was then cooled to 70°C. SM made without antibiotics was prewarmed to 42°C. The stock agarose solution was then diluted to 0.4% in the prewarmed SM. The SM and agarose mixture was then mixed with ethanol (0.05% final solution) and methanol (0.05% final solution). Then, 11.5 ml of the final mixture was poured into 100 mm by 15 mm petri dishes (Fisherbrand), which were allowed to dry with the lids off in a laminar flow hood for 1 h.

Parasites in mid-log-phase growth (1 × 10^6^ to 7 × 10^6^ cells/ml) were counted, harvested, and concentrated to 2 × 10^7^ cells/ml, and 5 μl of concentrated parasites were placed on the SoMo plate. Plates were allowed to incubate at room temperature for 10 min and were then wrapped in parafilm and placed at 28°C and 3% CO_2_. SoMo plates were moved to 28°C and 0% CO_2_ 24 h postplating.

### Chemotaxis assays.

Chemotaxis assays were developed based on reference 27. SoMo plates without antibiotics, T. brucei, and E. coli were prepared as described above. T. brucei was inoculated 1.5 cm from the center of the SoMo plate, and E. coli or the sample being tested was inoculated 2.0 cm from the center on the same horizontal axis (Fig. 2I), opposite the T. brucei. At 120 h postplating, the number of projections that had entered a 2-cm-diameter circle centered on the test sample location were counted. A chemotaxis index was calculated for each sample by subtracting the average number of projections entering the circle for the control condition with T. brucei alone from the average number of projections in samples plates, and that value was divided by the average number of projections in the sample plus the average number of projections in the control. Error bars represent the standard error of the mean, and unpaired two-tailed *t* tests were used to determine significance compared to the T. brucei-alone control condition.

Sterilized 1-cm-diameter Whatman filter discs were used for the filter paper conditions. For the E. coli on-lid conditions, 9 ml of a 1.0% SM and agarose solution was plated on the lid of the SoMo plate and allowed to dry in the same manner as the SoMo plate. E. coli was then plated 2 cm from the center on the lid of the plate. The lid was placed on the plate such that the E. coli on the lid and T. brucei on the plate aligned on the same horizontal axis. For the 0.2-μm filter experiment, 0.2 μm GNWP nylon membranes from Millipore were used. Because the 0.2-μm filters had a slightly larger diameter than the standard 2-cm diameter used in the other chemotaxis experiments, the number of projections that contacted the 0.2-μm filter were counted and compared under each condition.

For conditions in which E. coli cell lysate or formaldehyde-treated E. coli was tested, the number of bacterial cell equivalents that would have been present 4 days postplating, as determined in our bacterial growth curve, was used. To generate hypotonically lysed E. coli, 1 × 10^11^ cells/ml from a log-phase overnight culture in LB were centrifuged at 8,000 rpm for 10 min at 4°C. The supernatant was replaced with sterile MilliQ water. The sample was centrifuged again under the same conditions as above, and the supernatant was again replaced with water and lysozyme. The sample was then sonicated on ice 6 times for 10 s and then spun at 4,000 rpm for 10 min to pellet the cell debris. The supernatant was filter-sterilized through a 0.2-μm filter. For boiled E. coli lysate, 1 × 10^11^ cells/ml from a log-phase overnight culture in LB were centrifuged at 8,000 rpm for 10 min at 4°C, and the supernatant was replaced with sterile MilliQ water. The sample was then boiled for 10 min. Finally, for formaldehyde-treated E. coli, 1 × 10^11^ cells/ml from a log-phase overnight culture in LB were centrifuged at 8,000 rpm for 10 min at 4°C and washed in 1× phosphate-buffered saline (PBS), and the PBS was replaced with 4% paraformaldehyde. Tubes with formaldehyde-treated E. coli were rotated at 4°C for 10 min, washed 3 times in 1× PBS, and spun down and resuspended in the appropriate volume of 1× PBS.

### Time-lapse video analysis.

SoMo assays were performed as described above. At 24 h postplating, SoMo plates were inverted and placed on a ring stand over a light box. Still photographs were taken every 30 min for 162 to 185 h with a Brinno TLC200 Pro camera positioned above the inverted plate. Camera settings were configured to compile still images into an .avi file with a playback speed of 10 frames/second. The .avi movies were converted into stacks using Fiji version 1.0 (56), and the segmented-line tool was used to measure the distance each projection moved over time.

### Nonlinear regression analysis.

A nonlinear regression program was written in MATLAB to model the speed versus time data for the projections in the time-lapse videos. The “fit nonlinear regression model” feature in MATLAB was used to create models for a piecewise function that changed from a line with zero slope to a linear slope, an exponential function, and a linear function to find the best fit for the data.

### Individual cell motility analysis.

For Fig. 4, SoMo assays were performed as described above using a population of trypanosomes in which 10% GFP-tagged 29-13 cells were mixed with untagged cells and inoculated on the SoMo plate. Projections that came near enough to E. coli to begin moving toward it were placed in the “attraction” category, and those that did not were placed in the “no attraction” category. The tips of these projections were then imaged on a Zeiss Axiovert 200 M inverted microscope at ×20 magnification under bright-field microscopy. Movies (30 s each) of fluorescent cells in these same projections were then captured at 30 frames per second with Adobe Premiere Elements 9 using ×20 magnification under fluorescence microscopy. Fluorescent cells were tracked using a T. brucei-specific cell-tracking algorithm developed in MATLAB (32), and the resulting mean-squared displacement and curvilinear and straight-line velocities were calculated as described previously (57). Linearity is calculated as the ratio of straight-line velocity to curvilinear velocity. We only considered cells that were in focus for a minimum of 300 consecutive frames out of 900.

### Time course analysis assessing projection movement simultaneously with individual cell motility.

For the time course analyses in Fig. 5, SoMo assays using 1% GFP-tagged cells mixed with untagged cells were performed as described above. At each time point indicated, SoMo plates were photographed using a Fujifilm FinePix JZ250 digital camera, tips of projections were imaged on a Zeiss Axiovert 200 M inverted microscope as described above, and a 30-s video of GFP-expressing cells was captured as described above for each projection. This analysis was done for 11 projections each for T. brucei alone and T. brucei plus E. coli.

### Projection curvature calculation.

Bright-field images at ×20 magnification were acquired for the tip of projections. A straight line of 3-inch standard length was used to measure the angle of curvature of the tip of projections. One end of the standard was placed tangent to the peak of the projection tip, and a straight line was drawn at the other end, perpendicular to the standard until it intersected with the projection. The interior angle was then calculated and assigned as the angle of curvature (Fig. S2C).

### Data availability.

Source code for the MATLAB modeling used to create Fig. 3D, Fig. S3D, Table 2, and the speed versus time table in Table S1 can be found at https://gist.github.com/614a9d210af934b2cedcc3a76f1b66f1.git. The data in this paper will be made fully available without restriction.

10.1128/mSphere.00685-20.10 MOVIE S6 Time-lapse video of a projection impacting an E. coli colony. Still images were taken every 1 s at ×5 magnification using AxioVision 4.7.2 (12-2008) and played back at 5 frames per second. Download Movie S6, AVI file, 10.6 MB. Copyright © 2020 DeMarco et al. 2020 DeMarco et al. This content is distributed under the terms of the Creative Commons Attribution 4.0 International license.
