# Supplementary figures and images for: Identification of Positive Chemotaxis in the Protozoan Pathogen Trypanosoma brucei
Source: mSphere. 2020 Aug 12;5(4):e00685-20. doi: 10.1128/mSphere.00685-20 (PMC7426175; doi:10.1128/mSphere.00685-20)

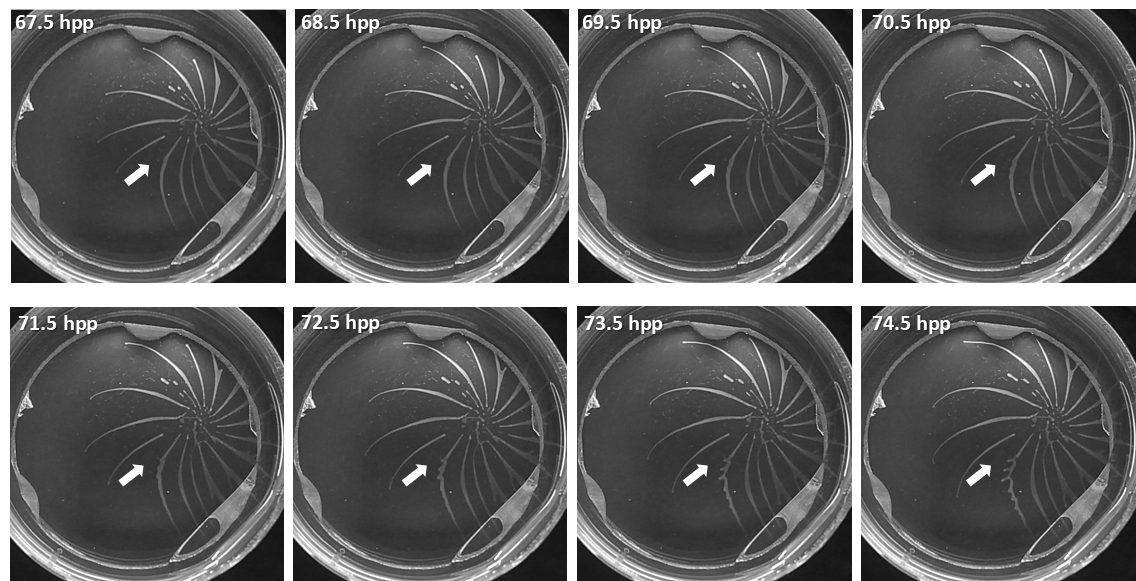

Supplement: FIG S1 [file mSphere.00685-20-sf001.tif]

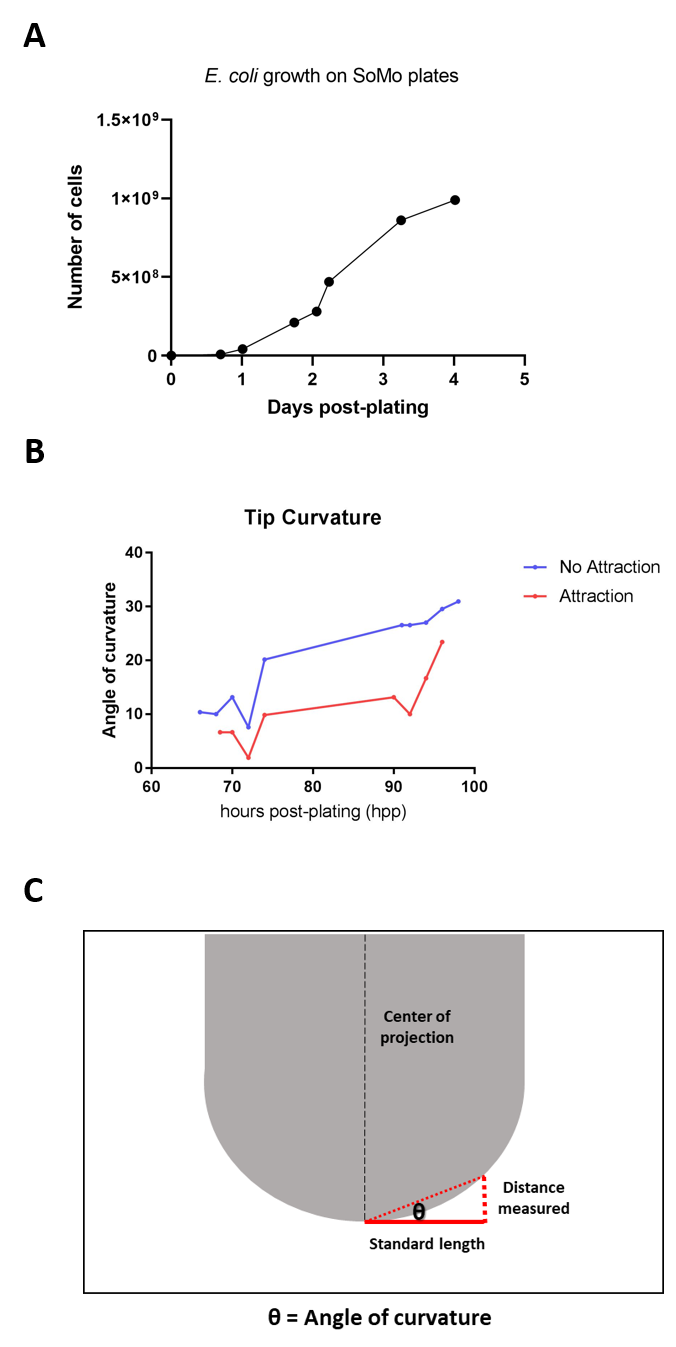

Supplement: FIG S2 [file mSphere.00685-20-sf002.tif]

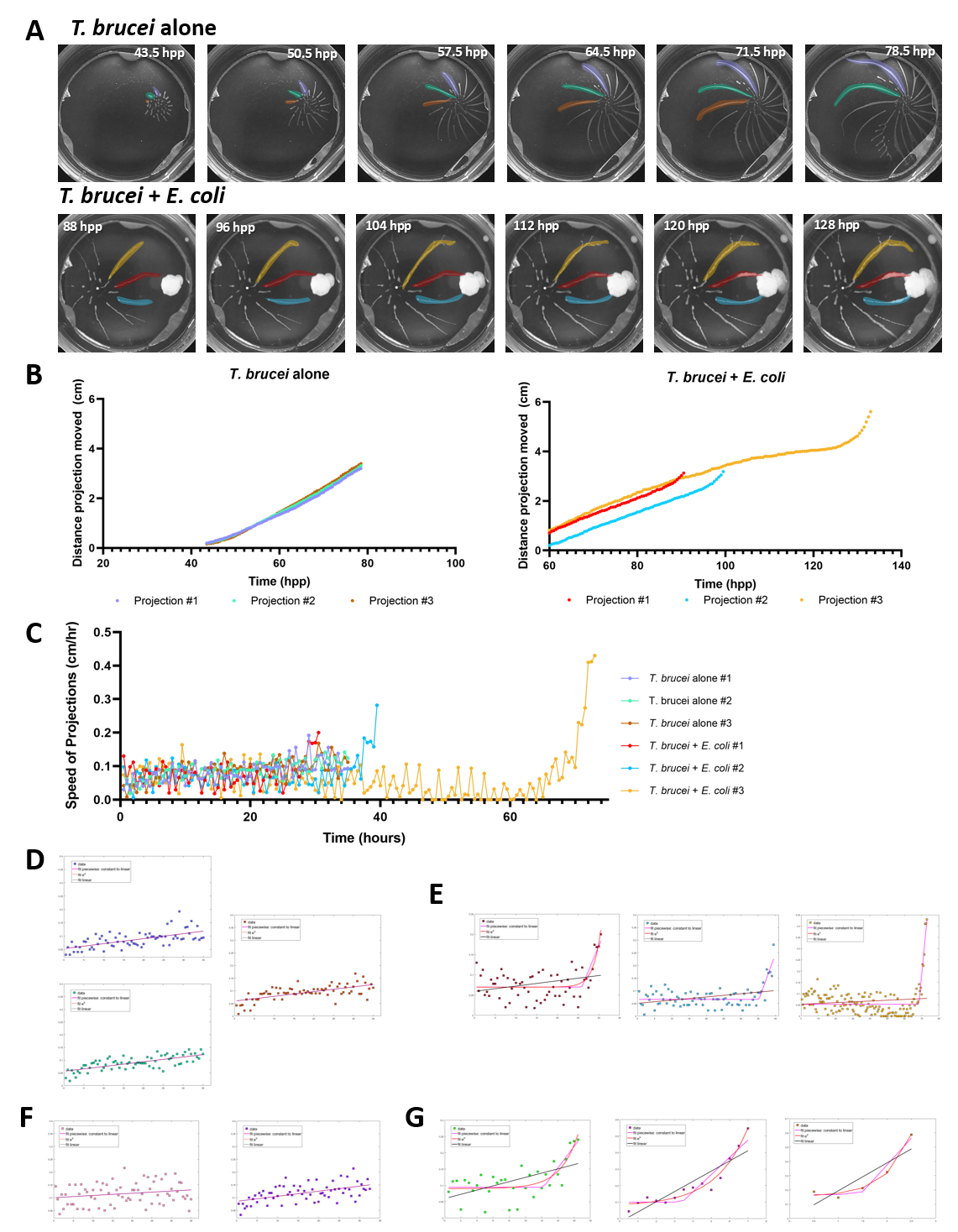

Supplement: FIG S3 [file mSphere.00685-20-sf003.tif]
