# Supplementary material for: Identification of Positive Chemotaxis in the Protozoan Pathogen Trypanosoma brucei
Source: mSphere. 2020 Aug 12;5(4):e00685-20. doi: 10.1128/mSphere.00685-20 (PMC7426175; doi:10.1128/mSphere.00685-20)
Supplement: TABLE S1 [file mSphere.00685-20-st001.docx]

| **Distance vs. Time graphs** | | |
| --- | --- | --- |
| Projection | Linear regression:  y = ax + b | Quadratic regression  y = ax^2^ + bx + c |
| *T. brucei* alone 1 | y = 0.0868x – 3.782  R² = 0.9898 | y = 0.0009x^2^ – 0.0277x – 0.387  R² = 0.9995 |
| *T. brucei* alone 2 | y = 0.0906x – 3.9619  R² = 0.9939 | y = 0.0007x^2^ – 0.0008x – 1.2546  R² = 0.9996 |
| *T. brucei* alone 3 | y = 0.96x – 4.2688  R² = 0.9941 | y = 0.0007x^2^ + 0.0049x – 1.5682  R² = 0.9992 |

| **Speed vs. Time graphs** | | | |
| --- | --- | --- | --- |
| Projection | Piece-wise function:  Constant to a line  For x ≤ k, y = a  For x > k, y = bx + c | Exponential regression  y = a e^bx^ + c | Linear regression  y = ax + b |
| *T. brucei* alone 1 | For x ≤ 0, y = 0.044  For x > 0, y = 1.87e^-3^ x + 0.0527  R^2^ = 0.378 | y = 5.83 e^3.19e-4 x^ – 5.77  R^2^ = 0.368 | y = 1.87e^-3^ x + 0.0527  R^2^ = 0.378 |
| *T. brucei* alone 2 | For x ≤ 0, y = 0.044  For x > 0, y = 1.87e^-3^ x + 0.0561  R^2^ = 0.491 | y = 6.80 e^2.73e-4 x^ – 6.75  R^2^ = 0.491 | y = 1.87e^-3^ x + 0.0561  R^2^ = 0.491 |
| *T. brucei* alone 3 | For x ≤ 0, y = 0.0437  For x > 0, y = 1.87e^-3^ x + 0.0594  R^2^ = 0.402 | y = 10.61 e^1.75e-4 x^ – 10.55  R^2^ = 0.402 | y = 1.87e^-3^ x + 0.0594  R^2^ = 0.402 |
| *T. brucei* + *E. coli* 1 | For x ≤ 25.92, y = 0.0698  For x > 25.92, y = 0.0248x – 0.574  R^2^ = 0.417 | y = 9.58e^-9^ e^0.541 x^ + 0.0695  R^2^ = 0.439 | y = 1.30e^-3^ x + 0.0592  R^2^ = 0.0701 |
| *T. brucei* + *E. coli* 2 | For x ≤ 35.27, y = 0.0659  For x > 35.27, y = 0.0377x – 1.265  R^2^ = 0.568 | y = -21.47 e^-5.88e-5 x^ + 21.52  R^2^ = 0.11 | y = 1.26e^-3^x + 0.0503  R^2^ = 0.11 |
| *T. brucei* + *E. coli* 3 | For x ≤ 68.35, y = 0.0525  For x > 68.35, y = 0.0805x – 5.449  R^2^ = 0.684 | y = -36.44 e^-9.79e-6 x^ + 36.49  R^2^ = 0.00495 | y = 3.57e^-4^x + 0.0526  R^2^ = 0.00496 |
